# Ecophysiology of neotropical amphibians and reptiles: lessons learned from Colombia

**DOI:** 10.1242/bio.059959

**Published:** 2023-09-21

**Authors:** Gustavo A. Agudelo-Cantero, Erika X. Cruz-Rodríguez, Faride Lamadrid-Feris, Jesús E. Ortega-Chinchilla

**Affiliations:** ^1^ Department of Physiology, Institute of Biosciences, University of São Paulo. Rua do Matão 101, Travessa 14, 05508-090 São Paulo, SP, Brazil; ^2^ Grupo de Herpetología, Eco-Fisiología y Etología, Departamento de Biología, Universidad del Tolima, Cl 42 # 1-02 Barrio Santa Helena Parte Alta, 730006299 Ibagué, Colombia; ^3^ Department of Biology, Faculty of Basic Sciences, Universidad del Atlántico, Cra 30 # 8-49 Puerto Colombia, 080001 Barranquilla, Colombia; ^4^ Laboratorio de Fisiología, Genómica y Transcriptómica adscrito al grupo CINBIN, Escuela de Biología, Universidad Industrial de Santander, Carrera 27 – Calle 9, Ciudad Universitaria, 680002 Bucaramanga, Colombia; ^5^ Departamento de Ciencias Básicas, Unidades Tecnológicas de Santander. Calle de los Estudiantes # 9–82, Ciudadela Real de Minas, 680005 Bucaramanga, Colombia

**Keywords:** Ecological physiology, Herpetology, Early-career researchers, Latin America

## Abstract

Ecophysiology and herpetology share a close historical relationship, but earlier work at the interface of these disciplines was carried out in temperate regions. Tropical regions like the Neotropics exhibit the highest species richness for amphibians and reptiles, but the pace for ecophysiological research on tropical herpetofauna has been slower relative to temperate counterparts. We are a group of early-career, Latin American researchers interested in the physiological diversity exhibited by neotropical herpetofauna. As such, we have engaged in the organization of the Symposium on the Ecophysiology of Neotropical Amphibians and Reptiles (ECOPHYSHERP) to integrate the scientific community interested on these topics. ECOPHYSHERP has been held three times already within the Colombian Congress of Herpetology, and collectively it has hosted >60 contributions from researchers at 26 institutions and eight countries. Participation has been diverse in terms of gender, age, and career stage, but most participants have been young undergraduate biology students. This generation of early-career researchers is producing excellent research in a broad range of topics, but difficulties to convert this research into scientific publications may exist. Identifying and contributing in order to solve such problems are priorities for this organizing committee, and also our endeavours towards ECOPHYSHERP 4.0 in Santa Marta in 2025.

## Teaser

**Summary:** We present progress in the ecological physiology of neotropical amphibians and reptiles through the lenses of our specialized symposium held in Colombia, and highlight the fundamental role of early-career herpetologists.

## Introduction

Ecological physiology (or ecophysiology), the study of physiological diversity in relation to the environments in which organisms live, arose at the interface of comparative physiology and natural history and consolidated as a separate field around the 1940s (Feder et al., 1987). Since its origins, ecophysiology has historically benefited from mutualistic relationships with taxonomic-based disciplines such as herpetology (the study of the biology of amphibians and reptiles). For instance, prominent ecophysiologists who have stemmed from George A. Bartholomew's legacy (one of the fathers of ecophysiology), such as P. Licht, V. H. Shoemaker, M. E. Feder, A. F. Bennet, and H. B. Lillywhite, among others (Bennett and Lowe, 2005), have been particularly interested in amphibians and reptiles. Similarly, renowned herpetologists such as C. Gans, P. E. Hertz, R. B. Huey, and A. S. Rand, among others, have made significant contributions to ecophysiology. The eventual encounter of these groups of researchers in the second half of the 20th century brought ecophysiology and herpetology closer and produced numerous publications, many of them in herpetological journals (e.g. Copeia, currently Ichthyology & Herpetology). This fruitful interaction occurred first in the USA, Canada, Europe, and Australia, and amphibian and reptile species (herpetofauna) being studied back then were mainly native to temperate regions. Thus, the herpetofauna of tropical zones remained comparatively less studied, from an ecophysiological perspective, until around the 1980s.

As for many organisms, the herpetofauna species richness is highest in the Neotropics (Bolaños et al., 2008; Uetz, 2023), where six out of 17 megadiverse countries – known to harbor up to 80% of Earth's biodiversity – are located (Mittermeier et al., 1997). Among these megadiverse countries, Colombia stands out for its location in the northernmost part of South America, where trans- and cis-Andean lineages converge. Heavily influenced by three cordilleras of the northern Andes, Colombia exhibits broad climatic variation and vegetation changes over relatively small areas (Marchant, 2008). For example, the Sierra Nevada de Santa Marta – an isolated coastal massif in northern Colombia – reaches a maximum elevation of ∼5700 m.a.s.l. just 46 km from the Caribbean coast, displays a 23°C change in annual average temperature, and encompasses six different biomes across elevation (Sesana, 2006). In addition, the less populated grassland plains eastward of the Andes and the Amazonian rainforests in southeastern Colombia – which collectively comprise over half of the territory – contain the most pristine ecosystems in the country. Regarding the herpetofauna, Colombia harbors ∼10.4% (903) and ∼5.4% (652) of known global amphibian and reptile species richness, respectively (Frost, 2023; Uetz, 2023), in only 0.02% of the Earth's land surface. Then, Colombia's multidimensional diversity offers stimulating opportunities for ecophysiological research in the Neotropics.

Key events favored the interaction between herpetology and ecophysiology in Colombia during the 20th century. Based on the influence of such events on the establishment of a research program at this disciplinary interface, we have identified three periods. The first period – the exploratory phase (late 1950s – early 1970s) – was marked by field expeditions carried out during this time. Stebbins and Hendrickson (1959), for instance, were perhaps the first to report data on field body temperature of amphibians in Colombia. During 1965–1966, Victor H. Hutchison led an expedition that aimed specifically to collect data on temperature tolerances and respiratory physiology of Colombian herpetofauna (Hutchison, 1971), starting ecophysiological research at an atypical time for herpetology in the country (Ardila-Robayo, 2003).

Hutchison's pioneer work opened the door to the second period of the ecophysiology-herpetology interaction in Colombia, the developing phase (∼early 1970s – late 1990s). At first, ecophysiological research was mainly species-specific, so that certain species were studied in depth (e.g. *Hyla labialis* at that moment, now *Dendropsophus molitor*; Mahoney and Hutchison, 1969; Valdivieso and Tamsitt, 1974). In parallel, the integrative work led by Pedro M. Ruiz-Carranza, María C. Ardila-Robayo, John D. Lynch and others in their group (e.g. Lynch, 1980; Lynch et al., 1997) to explain patterns of herpetofauna diversity across elevation was influential for many Colombian herpetologists, including those interested in ecophysiology. The convergence of these biogeographical insights with hypothesis-driven ecophysiological studies at high elevation in Peru and Chile (e.g. Marquet et al., 1989; Sinsch, 1988) propitiated the third period – the consolidating phase (∼late 1980s – late 1990s) – for the Ecophysiology-Herpetology interaction. This period, fostered by the work of Horst Lüddecke and later by Carlos A. Navas (e.g. Navas, 1996, 1997), broadened the scope of ecophysiological research from a species-specific view to a more encompassing, comparative one. Interestingly, the prevalent theme throughout these periods was thermal biology, mirroring the international scenario for the field back then.

In the 21st century, the interest for the ecophysiology of the Colombian herpetofauna has been increasing, particularly in the last decade. A broad literature search on Web of Science, complemented by references known by authors of this Meeting Review, showed that 45 scientific articles featuring 124 authors were published from 2000–2022, and 91.1% out of these publications occurred only from 2010 (Table S1, Fig. S1). Thermal biology continued being the most studied theme in the assessed period, though topics such as ecotoxicology, amphibian skin defenses (microbiota and bioactive compounds), and aspects of water balance gained importance (Fig. S2A). Anurans were the most studied taxonomic group, followed by lizards, and turtles (Fig. S2B). Interestingly, distinct life stages have been studied for both anurans and lizards, whereas studies with turtles have concentrated on embryos and hatchlings. Other groups (e.g. urodeles, caecilians, snakes, crocodilians) remain comparatively less studied. In addition, species occurring in the Andean region have been the most studied, followed by species from the Caribbean region (Fig. S2C). Other regions (i.e. Pacific, Orinoco, and Amazon) remain poorly explored. Collectively, these analyses indicate that there is much room for the ecophysiology-herpetology interaction in Colombia and point out to groups and regions that deserve more attention.

Motivated by the exuberant diversity of the neotropical herpetofauna, the challenges it faces (e.g. climate change, habitat loss, infectious diseases, invasive species, pollutants, etc.), our passion for ecophysiological research, and our Colombian roots, we have engaged in the organization of the Symposium on the Ecophysiology of Neotropical Amphibians and Reptiles (ECOPHYSHERP). This symposium has been held within the Colombian Congress of Herpetology (main conference), organized by the Colombian Association of Herpetology, three times (Fig. S3): the first edition in Medellín (2016), the second edition in Bogotá (2018), and the third edition in Cali (2022). ECOPHYSHERP aims to consolidate as the platform for integrating the scientific community working at the interface of ecophysiology and herpetology in the neotropical region and put Colombia in the spotlight for collaboration opportunities in the country. In this Meeting Review we discuss the main themes that have emerged at ECOPHYSHERP through its three editions, the representation and diversity of its participants, and initiatives to foster the development of early-career researchers. Finally, we present some research gaps at this disciplinary integration to guide future research efforts both in the Colombian and neotropical contexts.

## Herpetological ecophysiology for the 21st century: emerging themes from ECOPHYSHERP

**Fig. 1.**
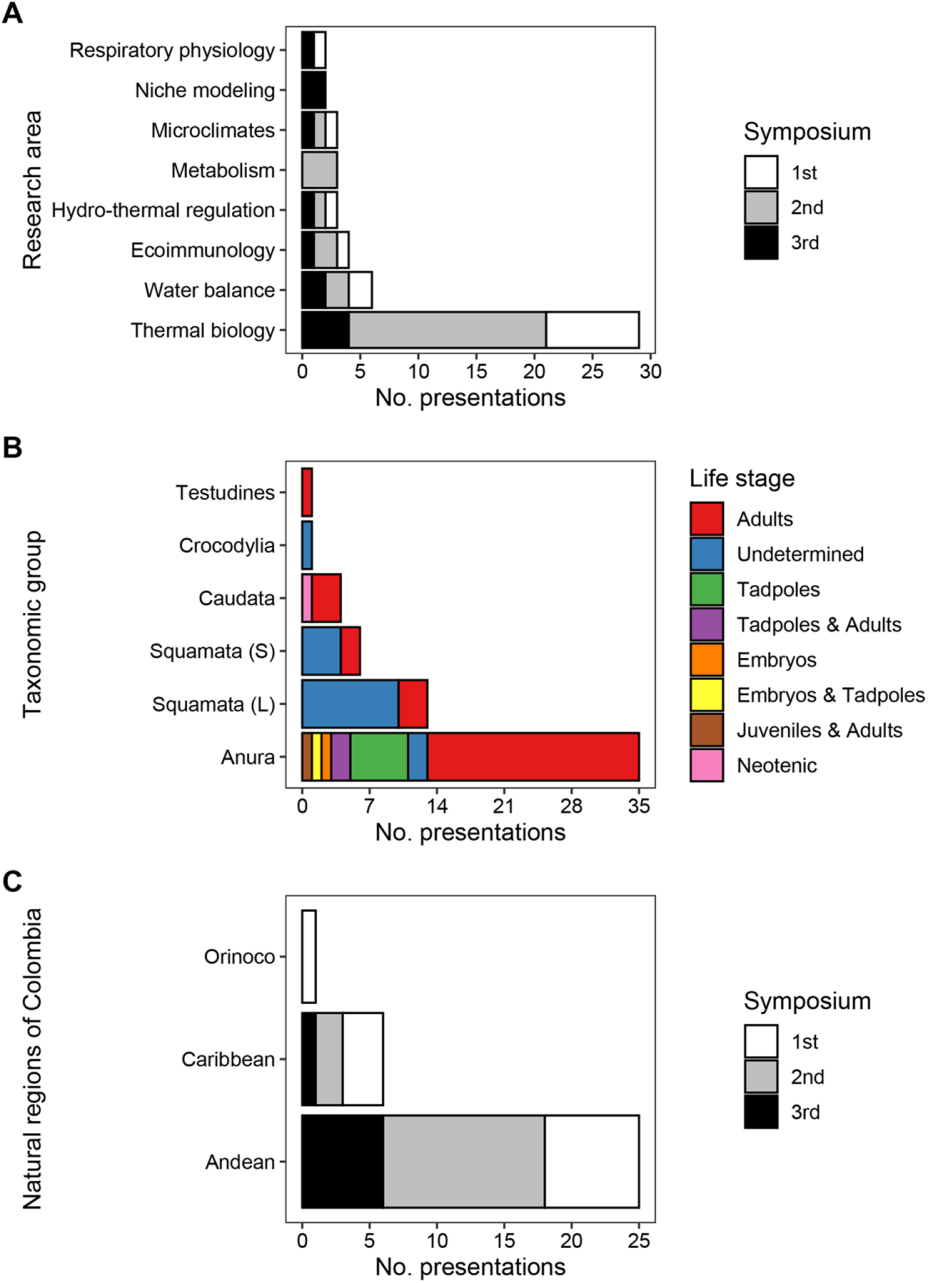
Emerging themes from ECOPHYSHERP by (A) research area, (B) taxonomic groups, and (C) natural regions of Colombia that were studied.

ECOPHYSHERP has seen a total of 65 presentations (eight keynote, 42 oral, 15 poster; Table S2) through its three editions, covering 20 research topics. Although categorizing research themes was difficult due to the interdisciplinary nature of many presentations, it is unambiguous that presentations at ECOPHYSHERP addressed a broader range of themes than scientific articles published in the last two decades (see Supplementary Material). Nevertheless, eight research themes accounted for 81% of all presentations, and thermal biology alone represented 45% (29; Fig. 1A).

That most presentations at ECOPHYSHERP were related to thermal biology was not surprising and reflects the history of the field in Colombia. We anticipated such a trend beforehand when started organizing the very first edition of the symposium. Therefore, we planned the keynote talk to be on water balance in anurans (see below), a much less studied topic in the country. After seeing that 47% of presentations at ECOPHYSHERP 1.0 dealt with aspects of thermal biology, we knew that the next editions of the symposium should contemplate at least one keynote talk on this topic. Then, Prof. Patricia Burrowes (University of Puerto Rico, San Juan, Puerto Rico) participated on ECOPHYSHERP 2.0 and presented her work across distinct tropical ecosystems to illustrate different responses of amphibians and reptiles to climate warming. During ECOPHYSHERP 3.0, Dr. Agustín Camacho (Estación Biológica de Doñana, Sevilla, Spain) questioned the use of distinct thermal traits to infer aspects of the vulnerability of reptiles to climate warming.

The enthusiasm of ECOPHYSHERP participants for thermal biology was evident by the broad scope of their investigations. The question of how some species thrive in extreme thermal environments has intrigued ecophysiologists for decades, and Valeria Ramírez Castañeda (Universidad de Los Andes, Bogotá, Colombia) addressed it nicely at ECOPHYSHERP 1.0 in her oral presentation on the heat tolerance of tadpoles and adults of the frog *Leptodactylus lithonaetes*. The thermal dependency of physiology, a classic topic in the field, was also well represented throughout all symposium editions, and here we highlight Juliana Poveda Cantini's talk (Universidad Nacional de Colombia, Bogotá, Colombia) at ECOPHYSHERP 2.0 on the effect of environmental temperature on the metabolism of the frog *Dendropsophus molitor* through its ontogeny. Individual physiology may also affect thermal biology, and that possibility was illustrated by Dr. Jesús E. Ortega-Chinchilla (University of São Paulo, São Paulo, Brazil) in his talk at ECOPHYSHERP 2.0 on the effects of gestation on the body temperature of the neotropical rattlesnake *Crotalus durissus*. The utility of thermal traits to infer aspects of the herpetofauna's vulnerability to warming was shown by Nathalia Suárez Ayala's talk (Universidad Militar Nueva Granada, Bogotá, Colombia), in which she mechanistically predicted the impact of several warming scenarios on activity time of the lizard *Anolis tolimensis* across distinct populations. Other addressed topics included thermoregulation in the field or laboratory conditions, impacts of the thermal environment on life history traits, and behavioral fever.

Other themes were comparatively less represented relative to thermal biology, but some emerging patterns are worth mentioning. In contrast with the recent literature, research on water balance and hydro-thermal regulation has been important throughout all ECOPHYSHERP editions. These areas have so much room to grow, as shown by Prof. Carlos A. Navas (University of São Paulo, São Paulo, Brazil) in his keynote talk during ECOPHYSHERP 1.0. Although the importance of water is considered obvious for amphibians (eight out of nine presentations on this topic used anurans or urodeles), the same is not for reptiles. Interestingly, Yelenny López Aguirre (Universidad del Quindío, Armenia, Colombia) showed at ECOPHYSHERP 3.0 that males and females of *Anolis antonii* display morphological variations across elevation that relate to both thermal and hydric gradients.

Another research theme that has been important at ECOPHYSHERP is Ecoimmunology. Interest in this area among Colombian ecophysiologists is likely very recent, considering it is still been assimilated in the literature. In this context, Leidy A. Barragán-Contreras (Universidad de Los Andes, Bogotá, Colombia) presented a talk during ECOPHYSHERP 1.0 about the effects of testosterone on morphometry and reproductive success of the caiman *Crocodilus crocodilus*. At ECOPHYSHERP 2.0, Dr. Faride Lamadrid Feris (University of São Paulo, São Paulo, Brazil) showed that experimentally-induced chronic stress probably has species-specific effects on anuran immunity, pointing out to the complexity of the relationship between stress and immunocompetence in anurans. To further stimulate this emerging theme, we invited Dr. Carla B. Madelaire (former Postdoctoral Researcher at University of Nevada, Las Vegas, USA) to participate in ECOPHYSHERP 3.0, where she presented a keynote talk on her work on the seasonality of stress hormones, metabolism, and reproductive activity of anurans from the Brazilian semiarid Caatinga.

A final comment concerns to taxonomic groups and natural regions of Colombia represented among ECOPHYSHERP presentations. Similar to the recent literature, anurans and lizards were the most studied groups by symposium participants (Fig. 1B). In both groups, however, the distribution of studied life stages was not as even as in the literature, for most works with amphibians focused on adults and most works with lizards did not specify or determine the life stage. Snakes and salamanders were also well represented in all ECOPHYSHERP editions. Interestingly, it seems that the Ramos' mushroom-tongue salamander (Plethodontidae: *Bolitoglossa ramosi*) is being used as a model organism to study aspects of water balance and hydro-thermal regulation in urodeles (see also below). For snakes, three out of six presentations used the thickhead ground snake (Dipsadidae: *Atractus crassicaudatus*) as model organism. Although common and abundant species may be good models for ecophysiological research, disentangling aspects of physiological diversity in both salamanders and snakes will require to expand efforts to other species. Regarding empirical research conducted within the Colombian territory and presented at ECOPHYSHERP (32 out of 65 presentations; Fig. 1C), the Andean region was by far the most represented (78%), followed by the Caribbean region (18.8%). These patterns mirror the published literature and are likely explained by the location of relevant research groups (see below). As in the literature, other Colombian regions (Orinoco, Pacific, and Amazon) remain underrepresented at ECOPHYSHERP.

## Representation and diversity

**Fig. 2.**
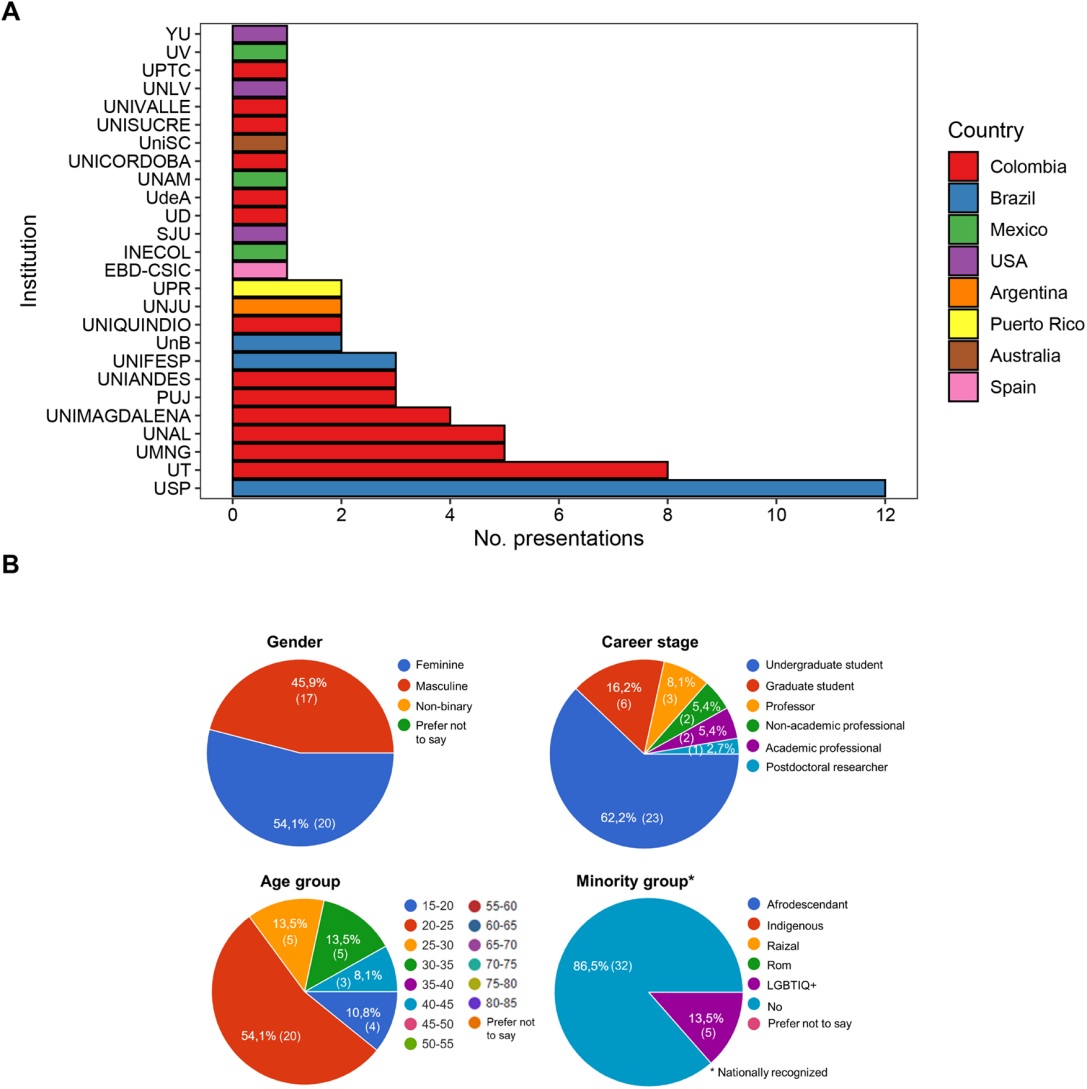
**Representation at ECOPHYSHERP.** (A) Participation by country and institution across all ECOPHYSHERP editions. USP, Universidade de São Paulo (Brazil); UT, Universidad del Tolima (Colombia); UMNG, Universidad Militar Nueva Granada (Colombia); UNAL, Universidad Nacional de Colombia (Colombia); UNIMAGDALENA, Universidad del Magdalena (Colombia); PUJ, Pontificia Universidad Javeriana (Colombia); UNIANDES, Universidad de Los Andes (Colombia); UNIFESP, Universidade Federal de São Paulo (Brazil); UnB, Universidade de Brasilia (Brazil); UNIQUINDIO, Universidad del Quindío (Colombia); UNJU, Universidad Nacional de Jujuy (Argentina); UPR, Universidad de Puerto Rico (Puerto Rico); EBD-CSIC, Estación Biológica de Doñana, CSIC (Spain); INECOL, Instituto Nacional de Ecología (Mexico); SJU, St. John's University (USA); UD, Universidad Distrital Francisco José de Caldas (Colombia); UdeA, Universidad de Antioquia (Colombia); UNAM, Universidad Nacional Autónoma de México (Mexico); UNICORDOBA, Universidad de Córdoba (Colombia); UniSC, University of the Sunshine Coast (Australia); UNISUCRE, Universidad de Sucre (Colombia); UNIVALLE, Universidad del Valle (Colombia); UNLV, University of Nevada Las Vegas (USA); UPTC, Universidad Pedagógica y Tecnológica de Colombia (Colombia); UV, Universidad Veracruzana (Mexico); YU, Yale University (USA). (B) Representation of participants and attendees in ECOPHYSHERP 3.0 according to gender, career stage, age group, and minorities.

Considering all symposium editions, participants represented 26 institutions (13 national, 13 international) in eight countries (Fig. 2A): Colombia, Brazil, Mexico, USA, Argentina, Puerto Rico, Australia, and Spain. Interestingly, 81% of all presentations were represented by participants from Colombia (55%) and Brazil (26%). Regarding participation per institution, the University of São Paulo (Brazil) was the most represented with 12 presentations, followed by the University of Tolima (Colombia) with eight presentations. These institutions host two research groups with long trajectory in the ecophysiology of herpetofauna in their respective countries, namely the Laboratory of Ecophysiology and Evolutionary Physiology (led by Prof. Carlos A. Navas at USP) and the Herpetology, Ecophysiology and Ethology Group (led by Prof. Manuel H. Bernal at UT). Other well-represented Colombian institutions in ECOPHYSHERP were: the Universidad Militar Nueva Granada (Ecotoxicology, Evolution, Environment and Conservation Group led by Prof. Nelsy R. Pinto-Sánchez); the National University of Colombia (Evolutionary Ecology and Morphology Group led by Prof. Martha L. Calderón-Espinosa); and the University of Magdalena (research led by Dr. Luis A. Rueda-Solano).

Information on diversity is available only for the third edition of ECOPHYSHERP. Participants and attendees were asked to fill out a form (in Spanish) to obtain their consent to collect, store, and disseminate data and/or photographs in connection with their participation at the symposium. Thirty-seven people, including all participants, from ten institutions answered the form and consented to share their data, which are summarized in Fig. 2B. Worth noting, most of respondents were women (54.1%). Regarding career stages, most respondents were undergraduate (62.2%) and graduate (16.2%) students. In addition, most respondents were under the age of 25 (64.9%). Importantly, 13.5% identified themselves as members of the LGBTIQ+ community.

## Relevant aspects to early-career researchers

In the context of the main conference, the best oral presentations by undergraduate students have been recognized in each ECOPHYSHERP edition. These presentations stood out for the excellence of the investigation as a process, from the identification of a problem or gap, the hypothesis/aims of the study, to the collection and interpretation of the resulting data. The speaker's domain of the study topic and the clarity of the presentation were also considered in our performance assessments. Besides the recognitions, issued by the Colombian Association of Herpetology, students also received relevant textbooks granted by the organization of the main conference.

Interestingly, all awardees were young women herpetologists working on aspects of amphibian water balance. Erika X. Cruz-Rodríguez (University of Tolima, Ibagué, Colombia) – ECOPHYSHERP 1.0 awardee, ECOPHYSHERP 3.0 co-organizer, and co-author of this Meeting Review – investigated the influence of relative humidity on the thermal preferences of *B. ramosi*. Raiza N. Castañeda (University of Magdalena, Santa Marta, Colombia) – ECOPHYSHERP 2.0 awardee – studied the influence of the hydric state on the locomotor performance of three treefrog species from the Colombian Caribbean. Laura A. Calvache-Naranjo (University of Tolima, Ibagué, Colombia) – ECOPHYSHERP 3.0 awardee – analyzed the impact of the hydric state on the locomotor performance of the Ramos' mushroom-tongue salamander.

Funding acquired for ECOPHYSHERP 3.0 was key to foster the development of early-career researchers. In coordination with the Society for the Study of Evolution – SSE (USA), we were able to grant 1-year SSE memberships to 19 participants and attendees (12 females – seven males; 14 undergraduate students – three graduate students – two academic professionals) from ten institutions. Moreover, we recognized the excellence of four presentations according to the career stages represented among symposium participants, i.e. undergraduate student, academic professional, master's student, and PhD student. Awardees (three females, one male) received a copy of the book ‘Evolution, 5th ed. (2022, Oxford University Press)’ by Douglas Futuyma and Mark Kirkpatrick.

## Concluding remarks and future directions

Ecophysiology and herpetology have a close historical relationship, and young Latin American biologists are finding an attractive niche at the intersection of these fields since early stages of their careers. Our analyses of the recent literature and our symposium indicate that the interest in the ecophysiology of amphibians and reptiles is growing in Colombia. Yet, the disparity between the number of research themes represented at ECOPHYSHERP and those in the literature indicates that early-career researchers likely face difficulties to convert symposium presentations into scientific publications. For instance, only seven works presented at ECOPHYSHERP were published from 2017–2022 (Table 1). We do not know what difficulties ECOPHYSHERP participants might encounter when trying to publish their research, but their nature is probably multifold, from language barriers, lack of training in academic writing, lack of financial support to cover publication fees, etc. Thus, a next step for us organizers of ECOPHYSHERP shall be to collect data from participants to promote problem-oriented activities (e.g. workshops, mentoring networks) that strengthen their skills and contribute to their career development.

**Table 1.**
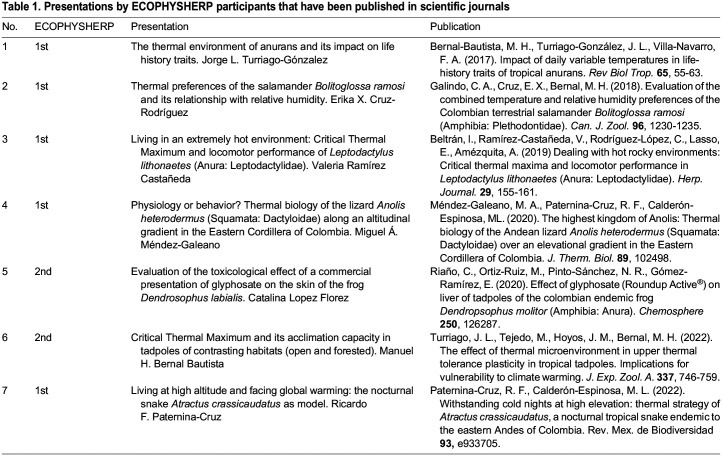
Presentations by ECOPHYSHERP participants that have been published in scientific journals

About scientific innovation and creativity, George A. Bartholomew once said that “…one can sometimes enhance the probability of making an innovative scientific contribution by transferring ideas and questions from one field of specialization to another, or from one taxonomic group to another…” (Bartholomew, 2005). Both the theoretical and methodological aspects of such a transfer shall consider the natural history of the study group to succeed, or otherwise there is a risk of “treating anurans as heliothermic lizards” (Navas et al., 2021). Nevertheless, we agree with Bartholomew's notion that scientific creativity is characteristic of young researchers, and ECOPHYSHERP has allowed us to witness that. In order to encourage this enthusiastic community, we present a non-exhaustive list of research themes, taxonomic groups, and Colombian regions that deserve further attention:

- Research themes: conservation physiology of the herpetofauna; neurophysiology of thermoregulation; physiological diversity of hydro-thermal regulation strategies; microclimate landscapes; temperature – disease interfaces.
- Taxonomic groups: anurans (embryos), salamanders, caecilians, snakes, turtles.
- Colombian regions: Pacific, Orinoco, Amazon.

## Supplementary Material

10.1242/biolopen.059959_sup1 Supplementary information
