# Supplementary material for: Ecophysiology of neotropical amphibians and reptiles: lessons learned from Colombia
Source: Biol Open. 2023 Sep 21;12(9):bio059959. doi: 10.1242/bio.059959 (PMC10537952; doi:10.1242/bio.059959)
Supplement: Supplementary information [file biolopen-12-059959-s1.pdf]

Table S1. References extracted from the Web of Science [((TS=(amphib* OR reptil* OR herpetolog* AND physiolog* OR ecophysiolog* AND Colombia)) AND AD=(Colombia)) AND DT=(Article); Dates searched: 2000-01-01 to 2022-12-31] on March 16, 2023, and filtered out by their scope fit within Ecophysiology. Few references known by authors of this meeting review and not included in the Web of Science were added.

Click here to download Table S1

Table S2. Scientific agenda of all ECOPHYSHERP editions

Click here to download Table S2

Dataset 1

Click here to download Dataset 1

Dataset 2

Click here to download Dataset 2

Dataset 3

Click here to download Dataset 3
